# Bmi1 Confers Resistance to Oxidative Stress on Hematopoietic Stem Cells

**DOI:** 10.1371/journal.pone.0036209

**Published:** 2012-05-11

**Authors:** Shunsuke Nakamura, Motohiko Oshima, Jin Yuan, Atsunori Saraya, Satoru Miyagi, Takaaki Konuma, Satoshi Yamazaki, Mitsujiro Osawa, Hiromitsu Nakauchi, Haruhiko Koseki, Atsushi Iwama

**Affiliations:** 1 Department of Cellular and Molecular Medicine, Graduate School of Medicine, Chiba University, Chiba, Japan; 2 Division of Stem Cell Therapy, Center for Stem Cell Biology and Regenerative Medicine, Institute of Medical Science, University of Tokyo, Tokyo, Japan; 3 RIKEN Research Center for Allergy and Immunology, Yokohama, Japan; 4 Japan Science and Technology Agency (JST), CREST, Tokyo, Japan; 5 ERATO, Chiyoda-ku, Tokyo, Japan; Emory University, United States of America

## Abstract

**Background:**

The polycomb-group (PcG) proteins function as general regulators of stem cells. We previously reported that retrovirus-mediated overexpression of *Bmi1*, a gene encoding a core component of polycomb repressive complex (PRC) 1, maintained self-renewing hematopoietic stem cells (HSCs) during long-term culture. However, the effects of overexpression of *Bmi1* on HSCs *in vivo* remained to be precisely addressed.

**Methodology/Principal findings:**

In this study, we generated a mouse line where *Bmi1* can be conditionally overexpressed under the control of the endogenous *Rosa26* promoter in a hematopoietic cell-specific fashion (*Tie2-Cre;R26Stop^FL^Bmi1*). Although overexpression of *Bmi1* did not significantly affect steady state hematopoiesis, it promoted expansion of functional HSCs during *ex vivo* culture and efficiently protected HSCs against loss of self-renewal capacity during serial transplantation. Overexpression of *Bmi1* had no effect on DNA damage response triggered by ionizing radiation. In contrast, *Tie2-Cre;R26Stop^FL^Bmi1* HSCs under oxidative stress maintained a multipotent state and generally tolerated oxidative stress better than the control. Unexpectedly, overexpression of *Bmi1* had no impact on the level of intracellular reactive oxygen species (ROS).

**Conclusions/Significance:**

Our findings demonstrate that overexpression of *Bmi1* confers resistance to stresses, particularly oxidative stress, onto HSCs. This thereby enhances their regenerative capacity and suggests that Bmi1 is located downstream of ROS signaling and negatively regulated by it.

## Introduction

Hematopoietic stem cells (HSCs) are defined as primitive cells that are capable of both self-renewal and differentiation into any of the hematopoietic cell lineages. Cell fate decisions of HSCs (self-renewal vs. differentiation) are precisely regulated to maintain their numbers and lifespan. Defects in these processes lead to hematopoietic insufficiencies and to the development of hematopoietic malignancies.

The polycomb-group (PcG) proteins play key roles in the initiation and maintenance of gene silencing through histone modifications. PcG proteins belong to two major complexes, Polycomb repressive complex 1 and 2 (PRC1 and PRC2). PRC1 monoubiquitylates histone H2A at lysine 119 and PRC2 trimethylates histone H3 at lysine 27 [1]. Of note, PcG proteins have been implicated in the maintenance of self-renewing stem cells [2]–[4]. Among PcG proteins, Bmi1, a core component of PRC1, plays an essential role in the maintenance of self-renewal ability of HSCs at least partially by silencing the *Ink4a/Arf* locus [5]–[8]. Bmi1 also maintains multipotency of HSCs by keeping developmental regulator gene promoters poised for activation [9]. Furthermore, Bmi1 has been implicated in the maintenance of the proliferative capacity of leukemic stem cells [5]. Consistent with these findings, levels of BMI1 expression in the human CD34^+^ cell fraction have been reported to correlate well with the progression and prognosis of myelodysplastic syndrome and chronic and acute myeloid leukemia [4], [10], suggesting a role of BMI1 in leukemic stem cells.

We previously reported that overexpression of *Bmi1* using a retrovirus maintains self-renewal capacity of HSCs and markedly expands multipotent progenitors *ex vivo*, resulting in an enhancement of repopulating capacity of HSCs after culture. Likewise, forced expression of *BMI1* was demonstrated to promote leukemic transformation of human CD34^+^ cells by *BCR-ABL* [11]. However, the effects of overexpression of *Bmi1* on hematopoiesis remained to be precisely addressed.

In this study, we generated mice overexpressing *Bmi1* in a hematopoietic cell-specific manner. We analyzed the effects of overexpression of *Bmi1* on hematopoiesis under steady state conditions as well as under multiple stresses. Our findings revealed a protective function for Bmi1 in HSCs from stresses, such as ROS, that usually limit the lifespan of HSCs.

## Results

### Generation of Mice Overexpressing *Bmi1* in Hematopoietic Cells

**Figure 1:**
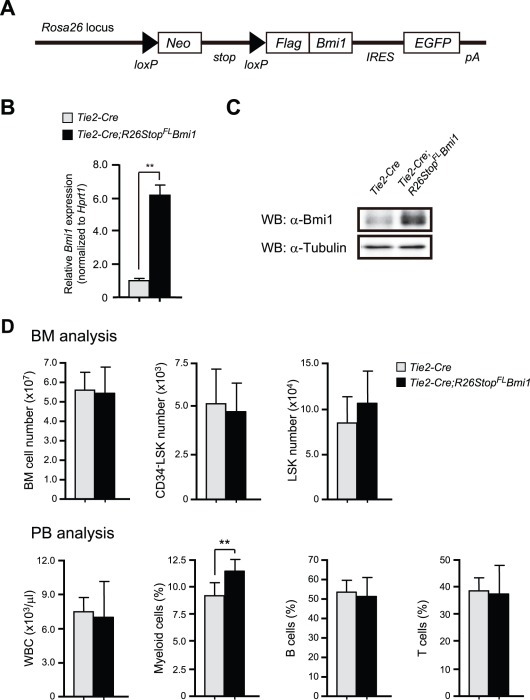
Generation of mice overexpressing *Bmi1* in hematopoietic cells. (A) Strategy for making a knock-in allele for *Bmi1* downstream of the *Rosa26* promoter. A *loxP*-flanked *neo^r^*-stop cassette followed by Flag-tagged *Bmi1*, an *frt*-flanked *IRES*-*eGFP* cassette, and a bovine polyadenylation sequence was knocked-in the *Rosa26* locus. (B) Quantitative RT-PCR analysis of *Bmi1* in BM LSK cells from *Tie2-Cre* and *Tie2-Cre;R26Stop^FL^Bmi1* mice. mRNA levels were normalized to *Hprt1* expression. Expression levels relative to that in *Tie2-Cre* LSK cells are shown as the mean ± S.D. (n = 3). (C) Western blotting analysis of Bmi1 in c-Kit^+^ BM cells from *Tie2-Cre* and *Tie2-Cre*;*R26Stop^FL^Bmi1* mice. α-tubulin was used as the loading control. (D) Hematopoietic analysis of 10-week-old *Tie2-Cre* and *Tie2-Cre;R26Stop^FL^Bmi1* mice. Absolute numbers of BM cells, CD34^-^LSK cells, and LSK cells in bilateral femurs and tibiae are presented as the mean ± S.D. (upper panels, *Tie2-Cre*; n = 7, *Tie2-Cre;R26Stop^FL^Bmi1*; n = 8). PB analysis of 10-week-old *Tie2-Cre* and *Tie2-Cre;R26Stop^FL^Bmi1* mice. White blood cell (WBC) counts and lineage contribution of myeloid, B, and T cells are shown as the mean ± S.D. (lower panels, *Tie2-Cre*; n = 7, *Tie2-Cre;R26Stop^FL^Bmi1*; n = 8). ** *p*<0.01.

To generate tissue-specific *Bmi1*-transgenic mice, we knocked a *loxP*-flanked *neo^r^*-stop cassette followed by Flag-tagged *Bmi1*, an *frt*-flanked *IRES*-*eGFP* cassette, and a bovine polyadenylation sequence into the *Rosa26* locus (**Figure 1A**). The obtained mice (hereafter referred to as *R26Stop^FL^Bmi1*) were crossed with *Tie2-Cre* mice [12] to drive *Bmi1* expression in a hematopoietic cell-specific manner. Quantitative RT-PCR analysis of bone marrow (BM) Lineage marker^-^Sca-1^+^c-Kit^+^ (LSK) cells confirmed 6-fold overexpression of *Bmi1* in *Tie2-Cre;R26Stop^FL^Bmi1* mice compared to the *Tie2-Cre* control mice (**Figure 1B**). Western blot analysis also verified overexpression of Bmi1 protein in BM c-Kit^+^ progenitor cells from *Tie2-Cre;R26Stop^FL^Bmi1* mice (**Figure 1C**).

### Steady State Hematopoiesis in *Tie2-Cre*;*R26Stop^FL^Bmi1* Mice

We first investigated the effect of overexpression of *Bmi1* on hematopoiesis in a steady state. Unexpectedly, 10-week-old *Tie2-Cre;R26Stop^FL^Bmi1* mice did not exhibit any significant differences in the numbers of total BM cells, CD34^-^LSK HSCs, LSK cells, multipotent progenitors (MPPs), common myeloid progenitors (CMPs), granulocyte/macrophage progenitors (GMPs), megakaryocyte/erythroid progenitors (MEPs), or common lymphoid progenitors (CLPs) compared to the *Tie2-Cre* control mice (**Figure 1D** and **Figure S1A**). The number of white blood cells (WBC) in peripheral blood (PB) did not change upon forced expression of *Bmi1*. Only the proportion of PB Gr-1^+^/Mac-1^+^ myeloid cells in *Tie2-Cre;R26Stop^FL^Bmi1* mice was significantly higher than in the control mice, although the difference was not drastic (a difference of only about 2%) (**Figure 1D**). Furthermore, *Tie2-Cre;R26Stop^FL^Bmi1* mice did not show any significant differences in the numbers of total spleen cells, LSK cells in the spleen, total thymic cells, or CD4^+^CD8^-^, CD4^-^CD8^+^, or CD4^+^CD8^+^ cells in the thymus compared to the control mice (**Figure S1A**). These findings indicate that overexpression of *Bmi1* does not largely compromise differentiation of HSCs. We further analyzed the cell cycle status of CD34^-^LSK HSCs by Pyronin Y staining, but again did not detect any changes (**Figure S1B**). These results indicate that overexpression of *Bmi1* only slightly perturbs hematopoiesis under steady state conditions, suggesting that the level of endogenous Bmi1 is sufficient to repress the transcription of its target genes.

### Colony-forming Capacity of Hematopoietic Stem and Progenitor Cells Overexpressing *Bmi1*

**Figure 2:**
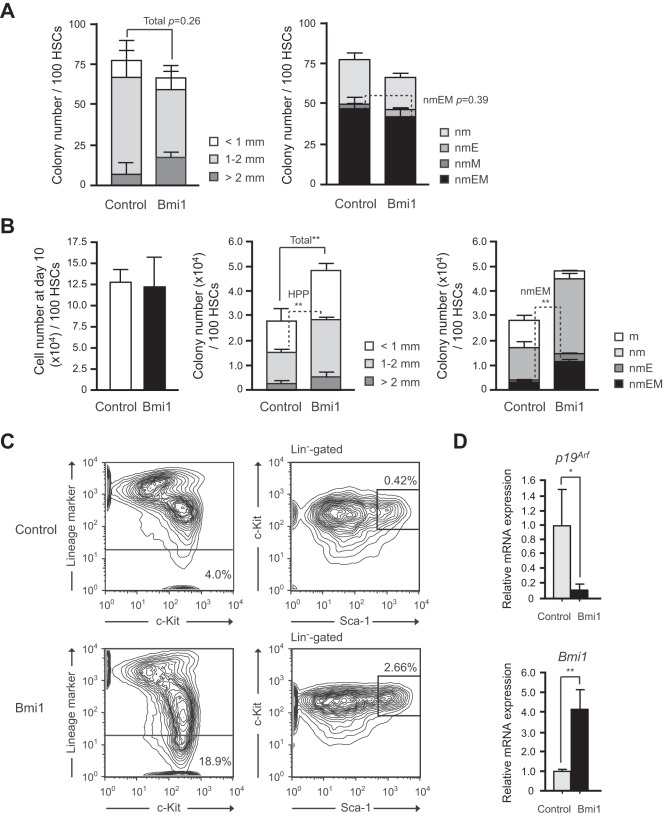
Effects of overexpression of *Bmi1* on HSCs *in vitro.* (A) Colony formation by HSCs isolated from *Tie2-Cre* (Control) and *Tie2-Cre*;*R26Stop^FL^Bmi1* (Bmi1) mice. Single CD34^-^LSK cells were sorted into 96-well microtiter plates containing the SF-O3 medium supplemented with 10% FBS and multiple cytokines (10 ng/ml SCF, 10 ng/ml TPO, 10 ng/ml IL-3, and 3 u/ml EPO) and allowed to form colonies. At day 14 of culture, the colonies were counted and individually collected for morphological examination. Absolute numbers of LPP and HPP-CFCs which gave rise to colonies with a diameter less and greater than 1 mm, respectively are shown as the mean ± S.D. for triplicate cultures (left panel). Absolute numbers of each colony types were defined by the composition of colonies (right panel). Colonies were recovered and examined by microscopy to determine colony types. Composition of colonies is depicted as n, neutrophils; m, macrophages; E, erythroblasts; and M, megakaryocytes. (B) Colony formation by HSCs cultured for 10 days. CD34^-^LSK cells from *Tie2-Cre* (Control) and *Tie2-Cre;R26Stop^FL^Bmi1* (Bmi1) mice were cultured in the SF-O3 serum-free medium supplemented with 50 ng/ml of SCF and TPO. At day 10 of culture, the cells were counted (left panel) and plated in methylcellulose medium to allow formation of colonies in the presence of 20 ng/ml SCF, 20 ng/ml TPO, 20 ng/ml IL-3, and 3 u/ml EPO. Absolute numbers of LPP and HPP-CFCs (middle panel) are shown as the mean ± S.D. for triplicate cultures. Absolute numbers of each colony type are shown in the right panel. (C) Flow cytometric analysis of CD34^-^LSK HSCs at day14 of culture. Representative flow cytometric profiles of LSK cells in cultures of CD34^-^LSK HSCs from *Tie2-Cre* (Control) and *Tie2-Cre;R26Stop^FL^Bmi1* (Bmi1) mice are depicted. The proportion of Lin^-^ and LSK cells in total cells are indicated. (D) Quantitative RT-PCR analysis of the expression of *p19^Arf^,* and *Bmi1* in *Tie2-Cre* (Control) and *Tie2-Cre;R26Stop^FL^Bmi1* (Bmi1) LSK cells. LSK cells were purified by cell sorting from CD34^-^LSK cultures in (C) at day 14 of culture. Each value was normalized to *Hprt1* expression and the expression level of each gene in control cells was arbitrarily set to 1. Data are shown as the mean ± S.D. for triplicate analyses. * *p*<0.05, **p<0.01.

We next evaluated the proliferative and differentiation capacity of *Tie2-Cre;R26Stop^FL^Bmi1* HSCs *in vitro*. Single CD34^-^LSK HSCs were clonally sorted into 96-microtiter plates with the medium supplemented with stem cell factor (SCF), thrombopoietin (TPO), interleukin-3 (IL-3), and erythropoietin (EPO) and allowed to form colonies. At day 14 of culture, the colonies were counted and individually collected for morphological examination. Both *Tie2-Cre* control and *Tie2-Cre;R26Stop^FL^Bmi1* HSCs gave rise to comparable numbers of high proliferative potential (HPP) and low proliferative potential (LPP) colonies with a diameter greater than and less than 1 mm, respectively (**Figure 2A**). The morphological analysis of colonies revealed that the number of colony-forming unit (CFU)-neutrophil/macrophage/erythroblast/megakaryocyte (nmEM) was also comparable between the two groups (**Figure 2A**). CFU-nmEM is a major subpopulation among CD34^−^LSK HSCs and its frequency is well correlated with that of functional HSCs [13]. These findings indicate that overexpression of *Bmi1* in freshly isolated CD34^-^LSK HSCs does not affect their colony-forming capacity or differentiation *in vitro*.

We previously reported that overexpression of *Bmi1* by retroviral transduction efficiently maintains hematopoietic stem and progenitor cells during long-term culture [7]. We re-evaluated the effect of forced expression of *Bmi1* using *Tie2-Cre;R26Stop^FL^Bmi1* HSCs. CD34^-^LSK cells were cultured for 10 days in a serum-free medium supplemented with SCF and TPO, a cytokine combination which supports the proliferation of HSCs and progenitors rather than their differentiation [14]. Although *Tie2-Cre;R26Stop^FL^Bmi1* HSCs did not show any growth advantage over the control (**Figure 2B**), the *Tie2-Cre;R26Stop^FL^Bmi1* HSC culture contained significantly more HPP-colony-forming cells (CFCs) and CFU-nmEM than the control (**Figure 2B**). Correspondingly, flow cytometric analysis revealed more LSK cells in the *Tie2-Cre;R26Stop^FL^Bmi1* HSC culture than in the control culture at day 14 (**Figure 2C**). There was no significant difference in the frequency of apoptotic cells between the control and *Tie2-Cre;R26Stop^FL^Bmi1* HSC cultures (**Figure S2A**). Of note, however, the *Tie2-Cre;R26Stop^FL^Bmi1* HSC culture contained a significantly higher proportion of LSK cells in the G_0_/G_1_ stage of cell cycle than the control (**Figure S2B**). These findings suggest that overexpression of *Bmi1* slows down cell cycle of immature hematopoietic cells in culture, leading to no growth advantages over the control cells in spite of an increase in immature progenitors in culture. As we reported previously, the *Ink4a/Arf* locus is a critical target of Bmi1 in HSCs [8]. Quantitative RT-PCR confirmed that *p19^Arf^* was closely repressed in transcription upon *Bmi1* overexpression (**Figure 2D**). These results support our previous finding that HSCs overexpressing *Bmi1* retain their self-renewal capacity better than the control HSCs under the culture stress.

### Overexpression of *Bmi1* Enhances Expansion of HSCs *ex vivo* and Protects HSCs During Serial Transplantation

**Figure 3:**
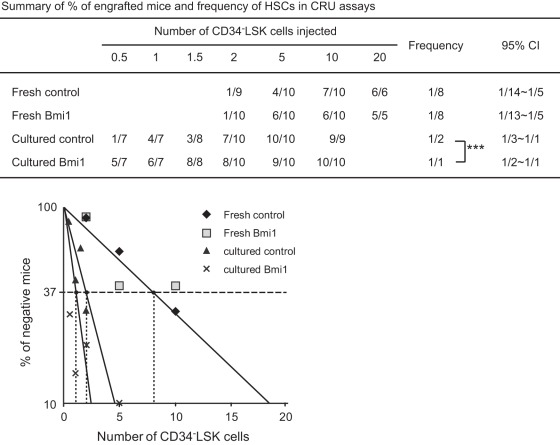
Overexpression of *Bmi1* enhances expansion of HSCs *ex vivo.* Competitive repopulating unit (CRU) assays using limiting numbers of CD34^-^LSK cells from *Tie2-Cre* (Control) mice and *Tie2-Cre;R26Stop^FL^Bmi1* (Bmi1) mice. Freshly isolated CD34^-^LSK cells were immediately used for BM transplantation, or CD34^-^LSK cells were cultured in the SF-O3 serum-free medium supplemented with 50 ng/ml SCF and TPO for 10 days, and then a fraction of the culture cells corresponding to the indicated number (0.5∼10) of initial CD34^-^LSK cells was subjected to BM transplantation. The test cells (CD45.2) were transplanted along with 2×10^5^ competitor BM cells (CD45.1) into CD45.1 recipient mice lethally irradiated at a dose of 9.5 Gy. Percent chimerism of donor cells in the recipient PB was determined at 16 weeks after transplantation. The mice with chimerism more than 1% in all three lineages (myeloid, B, and T cells) were considered successfully engrafted and the others were defined as negative mice. The frequency of HSCs was calculated using L-Calc software. The proportion of engrafted mice, frequency of functional HSCs, and the 95% confidence interval (CI) are summarized in the table and each data is plotted in the bottom panel. *** *p*<0.001.

HSCs are exposed to oxidative stress during long-term culture in 20% O_2_ [15]. In order to precisely determine the effect of overexpression of *Bmi1* on HSCs during culture, we next determined the frequency of functional HSCs contained in optimized serum-free culture by competitive repopulating unit (CRU) assay. We first transplanted limiting doses of fresh CD34^-^LSK cells from *Tie2-Cre* and *Tie2-Cre;R26Stop^FL^Bmi1* mice along with 2×10^5^ competitor BM cells. The frequency of long-term repopulating HSCs was 1 in 8 among fresh CD34^-^LSK cells from both *Tie2-Cre* and *Tie2-Cre;R26Stop^FL^Bmi1* mice (**Figure 3**). We then cultured CD34^-^LSK cells for 10 days in a serum-free medium supplemented with SCF and TPO in 20% O_2_. During the 10-day culture period, functional HSCs increased 4-fold (1 out of 2 CD34^-^LSK cells) in control CD34^-^LSK cells. Interestingly, overexpression of *Bmi1* established 2-fold better expansion of HSCs than the control during culture *ex vivo* (**Figure 3**). These findings are the first to show that Bmi1 has a role in the expansion of HSCs.

**Figure 4:**
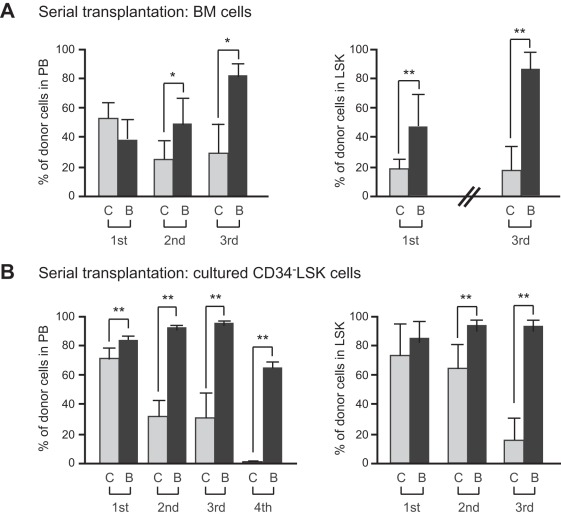
Overexpression of *Bmi1* protects HSCs during serial transplantation. (A) Serial transplantation of BM cells. BM cells (5×10^5^) from *Tie2-Cre* (denoted as “C”) and *Tie2-Cre;R26Stop^FL^Bmi1* (denoted as “B”) mice (CD45.2) along with 5×10^5^ competitor BM cells (CD45.2) were transplanted into CD45.1 recipient mice lethally irradiated at a dose of 9.5 Gy. For serial transplantation, BM cells were collected from all recipient mice at 12–20 weeks after transplantation and pooled together. Then, 5×10^6^ BM cells were transplanted into lethally irradiated recipient mice without competitor cells. Third and fourth transplantation were similarly performed using 5×10^6^ pooled BM cells. Percent chimerism of donor cells in the recipient PB and BM LSK cells was determined at 16 weeks post-transplantation. Results are shown as the mean ± S.D. (n = 6, 3^rd^ transplantation; n = 4). (B) Serial transplantation of cultured CD34^-^LSK cells. CD34^-^LSK cells were cultured in the SF-O3 serum-free medium supplemented with 50 ng/ml of SCF and TPO for 10 days. Then, the cells in culture corresponding to the 20 initial CD34^-^LSK cells were injected into a recipient mouse along with 2×10^5^ competitor BM cells (CD45.2) as described in (A) (n = 6, 4^th^ transplantation; n = 5). * *P*<0.05, ** *P*<0.01.

HSCs are exposed to various stresses including replicative and oxidative stresses during serial transplantation and eventually lose self-renewal capacity [16], [17]. We hypothesized that the effects of overexpression of *Bmi1* on HSCs would manifest under stressful conditions such as serial transplantations. Therefore, we performed competitive repopulation assays using 5×10^5^ fresh BM cells along with 5×10^5^ competitor BM cells (**Figure 4A**) or the total cells produced from 20 CD34^-^LSK cells after a 10-day culture period along with 2×10^5^ competitor BM cells (**Figure 4B**). The flow cytometric analysis of PB revealed little or no difference in the chimerism of donor cells between *Tie2-Cre* and *Tie2-Cre;R26Stop^FL^Bmi1* cells at 12 weeks after the primary transplantations. However, in the secondary and tertiary transplantations, the chimerism of *Tie2-Cre* cells significantly declined while that of *Tie2-Cre;R26Stop^FL^Bmi1* cells drasticaly increased. *Tie2-Cre* cells after 10-day culture failed to reconstitute hematopoiesis in the quaternary transplantation, while *Tie2-Cre;R26Stop^FL^Bmi1* cells still established robust repopulation (**Figure 4B**). The chimerism of donor cells in BM LSK cells mirrored the changes in the PB. These results clearly indicate that overexpression of *Bmi1* protects HSCs against the loss of self-renewal capacity during serial transplantation. The findings thus far suggest that overexpression of *Bmi1* confers stress resistance onto HSCs.

### Overexpression of *Bmi1* has no Impact on Radioprotection

DNA damage is intimately linked to stem cell aging. Heritable DNA damage accrued in stem cells leads to stem cell senescence or apoptosis, which over time can lead to the depletion of the stem cell pool and reduced regenerative capacity of stem cells [18]. Bmi1 is rapidly recruited to sites of DNA damage and is required for DNA damage-induced ubiquitination of histone H2A at lysine 119. Loss of Bmi1 leads to impaired repair of DNA double-strand breaks (DSBs) by homologous recombination [19], [20]. In glioblastoma multiforme (GBM) cells, Bmi1 was co-purified with DSB response proteins, such as ATM and the histone γH2AX, and non-homologous end joining (NHEJ) repair proteins. Of interest, BMI1 overexpression in normal neural stem cells enhanced ATM recruitment to the chromatin, the rate of γH2AX foci resolution, and resistance to radiation [21]. In order to understand the role of overexpressed Bmi1 in HSCs, we examined the radioresistance of HSCs by quantifying the number of γH2AX foci following genotoxic stress, a metric which reflects DNA DSBs.

**Figure 5:**
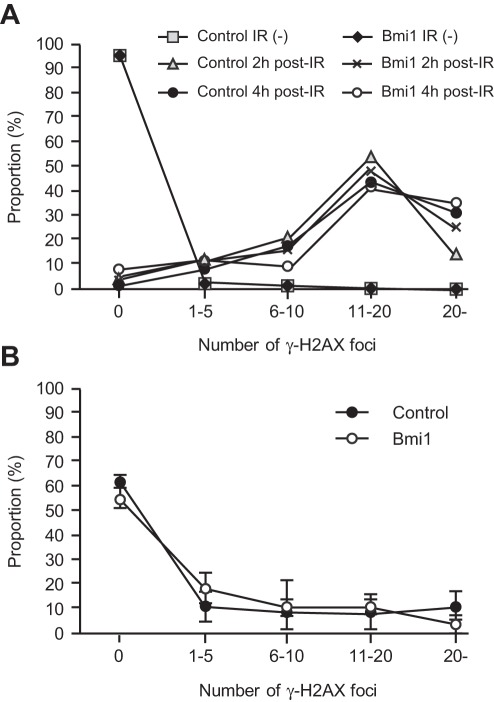
DNA damage response of *Tie2-Cre;R26Stop^FL^Bmi1* HSCs. (A) DNA damage response of CD34^-^LSK cells from *Tie2-Cre* (Control) and *Tie2-Cre;R26Stop^FL^Bmi1* (Bmi1) mice *in vitro*. Purified CD34^-^LSK cells were irradiated (IR) at a dose of 2 Gy. At 2 and 4 hours after irradiation, cells were stained with anti-γH2AX. Numbers of γH2AX foci expressed per cell are depicted. (B) DNA damage response of CD34^-^LSK cells from *Tie2-Cre* (Control) and *Tie2-Cre;R26Stop^FL^Bmi1* (Bmi1) mice *in vivo*. LSK cells were purified from the recipients of tertiary transplantation and stained with anti-γH2AX. Numbers of γH2AX foci expressed per cell are depicted as the mean ± S.D. (n = 3).

We purified CD34^-^LSK cells from *Tie2-Cre* and *Tie2-Cre*;*R26Stop^FL^Bmi1* mice and irradiated them at a dose of 2 Gy. At 2 and 4 hours after irradiation, cells were stained with anti-γH2AX. We expected rapid resolution of γH2AX by overexpression of *Bmi1*, but no significant difference was observed in the number of γH2AX foci between *Tie2-Cre* and *Tie2-Cre;R26Stop^FL^Bmi1* HSCs (**Figure 5A**). HSCs recovered from the recipients of tertiary transplantation did not show any difference in the number of γH2AX foci, either (**Figure 5B**). We then tested hematopoietic recovery after irradiation in mice. We irradiated recipient mice reconstituted with *Tie2-Cre* and *Tie2-Cre;R26Stop^FL^Bmi1* BM cells at a dose of 5 Gy, and monitored hematopoietic recovery for 4 weeks. The recovery of hematopoietic components in PB as well as BM LSK cells was comparable between the two groups (**Figure S3**). These findings suggest that overexpression of *Bmi1* does not afford an advantage to HSCs in their ability to resist genotoxic stress.

### Overexpression of *Bmi1* Confers Resistance to Oxidative Stress on HSCs

HSCs contain lower levels of reactive oxygen species (ROS) than their mature progeny in order to maintain their quiescent state. ROS reportedly act through p38 mitogen-activated protein kinase (MAPK) to limit the lifespan of HSCs [16], [22]. It has been demonstrated that prolonged treatment with the antioxidant *N*-acetyl-_L_-cysteine (NAC) or an inhibitor of p38 MAPK extends the lifespan of HSCs in serial transplantation assays, suggesting that oxidative stress is one of the major factors that affects HSC function during these assays [16], [17], [23]. Given that *Tie2-Cre;R26Stop^FL^Bmi1* HSCs retain self-renewal capacity during serial transplantation, overexpression of *Bmi1* may bestow a protective effect onto HSCs against oxidative stress.

**Figure 6:**
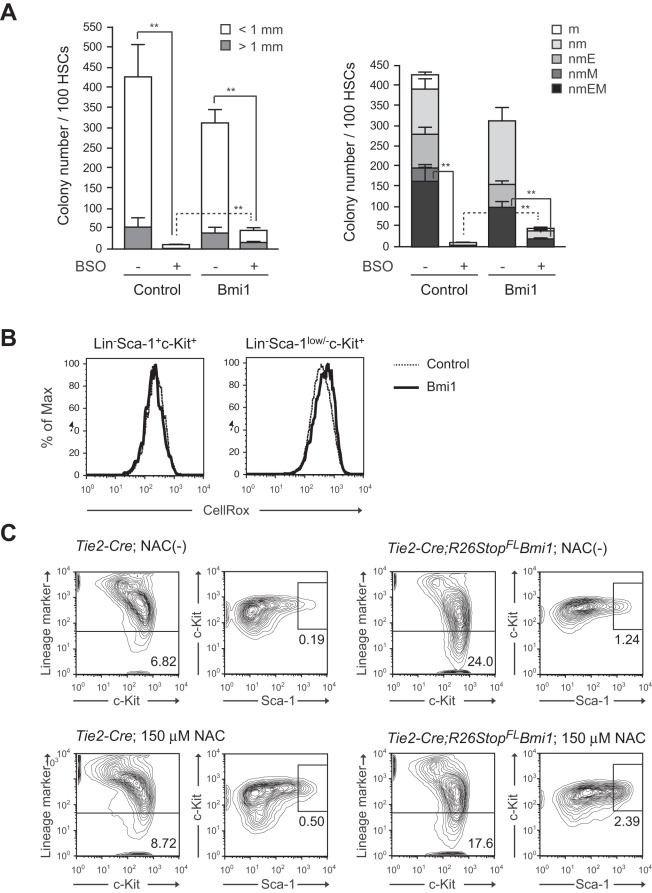
Overexpression of Bmi1 confers oxidative stress on HSCs. (A) Colony formation by HSCs cultured for 3 days. CD34^-^LSK cells from *Tie2-Cre* (Control) and *Tie2-Cre;R26Stop^FL^Bmi1* (Bmi1) mice were cultured in the SF-O3 serum-free medium supplemented with 50 ng/ml SCF, TPO and 0.05 mM of BSO. At day 3 of culture, the cells were plated in methylcellulose medium to allow formation of colonies in the presence of 20 ng/ml SCF, 20 ng/ml TPO, 20 ng/ml IL-3, and 3 u/ml EPO. Absolute numbers of LPP and HPP-CFCs (left panel) are shown as the mean ± S.D. for triplicate cultures. Absolute numbers of each colony types are shown in the right panel. Data are shown as the mean ± S.D. for triplicate analyses. Statistical analyses were performed on the total colony numbers (left panel) and nmEM colony numbers (right panel), respectively. **p<0.01. (B) Levels of ROS in cells overexpressing *Bmi1*. CD34^-^LSK cells from *Tie2-Cre* (Control) and *Tie2-Cre;R26Stop^FL^Bmi1* (Bmi1) mice were cultured in the SF-O3 serum-free medium supplemented with 50 ng/ml SCF and TPO. Representative flow cytometric profiles of LSK and Lineage marker^-^Sca-1^low/−^c-Kit^+^ cells in cultures at day 14 are depicted. (C) Effects of NAC on *Bmi1* culture. CD34^-^LSK cells from *Tie2-Cre* and *Tie2-Cre;R26Stop^FL^Bmi1* mice were cultured in the SF-O3 serum-free medium supplemented with 50 ng/ml SCF and TPO in the presence and absence of 150 µM NAC. Representative flow cytometric profiles of LSK cells in cultures at day 14 are depicted. The proportion of Lin^-^ and LSK cells in total cells are indicated.

To address this question, we cultured HSCs in the presence of buthionine sulfoximine (BSO), which depletes intracellular glutathione and thereby increases intracellular ROS levels. We found that highly purified CD34^-^LSK HSCs were susceptible to an increase in ROS levels because treatment with BSO significantly suppressed their growth and induced cell death (data not shown). After 3 days of BSO treatment, surviving cells were subjected to colony-forming assays. Both *Tie2-Cre* control and *Tie2-Cre;R26Stop^FL^Bmi1* HSCs cultured with BSO gave rise to significantly fewer colonies than HSCs cultured without BSO. Interestingly, *Tie2-Cre;R26Stop^FL^Bmi1* HSCs gave rise to a significantly more colonies than the control HSCs (**Figure 6A**). Notably, the number of HPP colonies was reduced 48-fold after treatment of control HSCs with BSO, but only 3-fold upon overexpression of *Bmi1*. The frequency of CFU-nmEM was also less perturbed following treatment with BSO in HSCs overexpressing *Bmi1*. These results indicate a role for Bmi1 in the resistance to oxidative stress.

Bmi1 regulates mitochondrial function by regulating the expression of a cohort of genes related to mitochondrial function and ROS generation. *Bmi1*-deficient cells have impaired mitochondrial function, which causes a marked increase in the intracellular levels of ROS [24]. Based on these observations, we then measured the intracellular ROS levels in CD34^-^LSK cells at day 14 of culture. Unexpectedly, overexpression of *Bmi1* did not affect the levels of ROS in either LSK HSCs/MPPs or Lin^-^Sca-1^low/−^c-Kit^+^ downstream progenitors (**Figure 6B**). Overexpression of *Bmi1* had no significant effect on the ROS levels even in the presence of BSO (**Figure S4**). Likewise, treatment of cells with the antioxidant NAC promoted cell growth and increased the proportion of LSK cells in both control and *Tie2-Cre;R26Stop^FL^Bmi1* culture similarly (**Figure 6C** and data not shown). These results indicate that an excess of Bmi1 does not regulate the generation or scavenging of ROS, but confers resistance to higher levels of ROS on HSCs through unknown mechanisms.

## Discussion

In this study, we generated a new mouse line where *Bmi1* can be conditionally overexpressed in a hematopoietic cell-specific fashion and analyzed the effect of overexpression of *Bmi1* in detail. Overexpression of *Bmi1* did not significantly affect steady state hematopoiesis, but it efficiently protected HSCs from stresses. Our findings suggest that overexpression of *Bmi1* confers resistance to stresses on HSCs, thereby augmenting their regenerative capacity.

Recent findings have established that the regulation of oxidative stress in HSCs is critical for the maintenance of HSCs. In this study, we demonstrated that overexpression of *Bmi1* protects HSCs from loss of self-renewal capacity at least in part by increasing the capacity of HSCs to resist oxidative stress. It has been reported that *Bmi1*-deficient mice have an increased level of intracellular ROS due to de-regulated expression of genes related to mitochondrial function and ROS generation [24], [25]. However, an excess of Bmi1 in this study had no effect on the levels of intracellular ROS. Thus, it is hypothesized that Bmi1 is negatively regulated downstream of the ROS signal and an excess of Bmi1 overcomes this negative regulation. Indeed, ROS reportedly primes *Drosophila* hematopoietic progenitors for differentiation and this process involves downregulation of PcG activity [26]. ROS signaling activates p38 and eventually releases the transcriptional repression of *p16^Ink4a^* and *p19^Arf^*, critical targets of Bmi1 [16]. Furthermore, recent studies including ours have revealed that PcG proteins are downregulated and dissociate from the *Ink4a/Arf* locus when cells are exposed to intra- or extracellular stress, including tissue culture- and oncogene-induced stress [27], [28]. Together, this accumulating evidence suggests that Bmi1 is dynamically regulated in response to oxidative stress, probably downstream of p38. Our preliminary data demonstrated that activated p38 directly phosphorylates Bmi1 *in vitro* (Oshima and Iwama., unpublished data). Thus, it is possible that p38, which is activated by oxidative stress, attenuates Bmi1 function via direct phosphorylation of Bmi1. How oxidative stress restricts the expression and function of Bmi1 is an important issue to be addressed.

Of note, the effect of Bmi1 overexpression in serial transplantation resembles that of overexpression of *Ezh2*, a gene encoding a core component of PRC2 [29]. Overexpression of PcG genes, *Bmi1* and *Scmh1*, also induces tolerance of cortical neurons to ischemia [30]. Thus, various cellular stresses may target PcG complexes to release transcriptional repression of PcG-regulated genes, such as tumor suppressor and developmental regulator genes, thereby affecting stemness. All these findings support the notion that enforcement of PcG function is a key for successful regenerative therapies.

Meanwhile, the role of PcG proteins in resistance to oxidative stress is also implicated in cancer. Expression of PcG proteins including *BMI1* and *EZH2* are often up-regulated in various cancers, particularly in their cancer stem cell fractions [31]. Interestingly, cancer stem cells in some tumors appear to be susceptible to ROS, similar to normal stem cells, and thus develop mechanisms to keep the levels of ROS low [32]. Interference of EZH2 function by the small-molecule histone methyltransferases inhibitor, DZNep, is reported to increase ROS levels in acute myeloid leukemia cells like in *Bmi1*-deficient mice [33]. Conversely, our findings in this study suggest that an excess of PcG proteins often observed in aggressive cancer could help cancer stem cells tolerate oxidative stress. In this regard, overexpression of PcG proteins could also be therapeutic targets in cancers including leukemia. Finally, no *Tie2-Cre;R26Stop^FL^Bmi1* mice developed hematological malignancies during the observation period, up to 18 months after birth. Only one recipient mice with *Tie2-Cre;R26Stop^FL^Bmi1* BM cells developed acute lymphocytic leukemia in the tertiary transplantation. These findings suggest that Bmi1 by itself is not sufficient to induce hematological malignancies.

## Methods

### Ethics Statement

All experiments using the mice were performed in accordance with our institutional guidelines for the use of laboratory animals and approved by the review board for animal experiments of Chiba University (approval ID: 21–150).

### Generation of Mice

To generate tissue-specific *Bmi1*-transgenic mice, we used the plasmid *R26Stop^FL^*, a modified version of pROSA26-1 with a *loxP*-flanked *neo^r^*-stop cassette, an *frt*-flanked *IRES*-*eGFP* cassette, and a bovine polyadenylation sequence [34]. We cloned a cDNA encoding a flag-tagged *Bmi1* upstream of the *IRES* sequence (*R26Stop^FL^Bmi1*). R1 ES cells were transfected, cultured, and selected as previously described [35]. For conditional expression of *Bmi1*, the *RosaStop^FL^Bmi1* mice were crossed with *Tie2-Cre* mice. C57BL/6 (CD45.2) mice were purchased from Japan SLC (Shizuoka, Japan). C57BL/6 mice congenic for the Ly5 locus (CD45.1) were purchased from Sankyo-Lab Service (Tsukuba, Japan). Mice were bred and maintained in the Animal Research Facility of the Graduate School of Medicine, Chiba University in accordance with institutional guidelines. This study was approved by the institutional review committees of Chiba University (approval numbers 21–65 and 21–150).

### Flow Cytometric Analysis and Cell Sorting

Mouse CD34^–^LSK HSCs were purified from BM of 8–12-week-old mice. Mononuclear cells were isolated on Ficoll-Paque PLUS (GE Healthcare). Cells were stained with an antibody cocktail consisting of biotinylated anti-Gr-1, Mac-1, interleukin (IL)-7Rα, B220, CD4, CD8α, and Ter119 monoclonal antibodies. The monoclonal antibodies were purchased from eBioScience or BioLegend. Lineage-positive cells were depleted with goat anti-rat IgG microbeads (Miltenyi Biotec) through an LS column (Miltenyi Biotec). Cells were further stained with Alexa Fluor® 647 or eFluor® 660-conjugated anti-CD34, phycoerythrin (PE)-conjugated anti-Sca-1, and phycoerythrin/Cy7 (PE/Cy7)-conjugated anti-c-Kit antibodies. Biotinylated antibodies were detected with allophycocyanin/Cy7 (APC/Cy7)-conjugated streptavidin. Dead cells were eliminated by staining with Propidium iodide (1 µg/ml, Sigma). Analysis and sorting were performed on a FACS Aria II (BD Bioscience).

### Cell Cycle Analysis

Fresh BM cells (1×10^7^, CD45.2) were transplanted into 8-week-old CD45.1 mice irradiated at a dose of 9.5 Gy without competitor cells. Four months later, BM mononuclear cells were isolated on Ficoll-Paque PLUS. Cells were stained with an antibody cocktail consisting of biotinylated anti-Gr-1, Mac-1, IL-7Rα, B220, CD4, CD8α, Ter119, and CD45.1 monoclonal antibodies. Cells were further stained with Alexa Fluor® 700-conjugated anti-CD34, pacific blue-conjugated anti-Sca-1, and APC-conjugated anti-c-Kit antibodies. Biotinylated antibodies were detected with APC/Cy7-conjugated streptavidin. Analysis was performed on a FACS Aria II. To analyze the cell-cycle status, cells were incubated with 1 µg/ml Pyronin Y (Sigma) at 37°C for 45 min with protection from light. Bulk sorted CD34^-^LSK cells were incubated in SF-O3 supplemented with 50 µM?2-β-mercaptoethanol, 0.2% BSA, 1% GPS, 50 ng/ml SCF, 50 ng/ml TPO for 10 days at 37°C in a 5% CO_2_ atmosphere. At day 10 of culture, the cell cycle profiles of culture cells were analyzed using an APC BrdU Flow Kit (BD Pharmingen). The cells were incubated with 10 µM BrdU at 37°C for 30 min and then stained with an antibody cocktail consisting of biotinylated anti-Gr-1, Mac-1, IL-7Rα, B220, CD4, CD8α, and Ter119 monoclonal antibodies. Cells were further stained with PE-conjugated anti-Sca-1, and PE/Cy7-conjugated anti-c-Kit antibodies. Biotinylated antibodies were detected with APC/Cy7-conjugated streptavidin. Analysis was performed on a FACS Canto II (BD Bioscience).

### Colony Assay

Colony assays were performed in methylcellulose-containing Iscove’s modified Dulbecco’s medium (Methocult M3234; Stemcell Technologies) supplemented with 20 ng/ml mouse SCF, 20 ng/ml mouse IL-3, 20 ng/ml human TPO, and 3 U/ml human EPO (Peprotech), and incubated at 37°C in a 5% CO_2_ atmosphere. The number of HPP- and LPP-colony-forming cells (CFCs), which generate a colony with a diameter ≥1 mm and <1 mm, respectively, were evaluated by counting colonies at day 10–14 of culture. Colonies were individually collected, cytospun onto glass slides, and subjected to Hemacolor (MERCK) staining for morphological examination. To evaluate the proliferative and differentiation capacity of *Tie2-Cre;R26Stop^FL^Bmi1* HSCs *in vitro*, single CD34^-^LSK HSCs were clonally sorted into 96-microtiter plates containing 100 µl SF-O3 (Sanko Junyaku) supplemented with 50 µM 2-β-mercaptoethanol, 10% FBS, 1% L-glutamine, penicillin, streptomycin solution (GPS; Sigma), 10 ng/ml mouse SCF, 10 ng/ml human TPO, 10 ng/ml mouse IL-3, and 3 unit/ml human EPO (PeproTech). At day 14 of culture, the colonies were counted and individually collected for morphological examination. To evaluate the tolerance of test cells against oxidative stress, CD34^-^LSK cells were cultured in the presence of DL-Buthionin-(*S,R*)-sulfoximine (BSO, Sigma) or *N*-Acetyl-_L_-cysteine (NAC, Sigma) for the indicated time periods, then subjected to colony assays or flow cytometric analyses.

### Serial Transplantation and CRU Assays

Fresh BM cells (5×10^5^, CD45.2) or 10-day cultured CD34^-^LSK cells (CD45.2) corresponding to 20 initial CD34^-^LSK cells were transplanted into 8-week-old recipient mice (CD45.1) irradiated at a dose of 9.5 Gy together with 5×10^5^ and 2×10^5^ BM competitor cells from 8-week-old CD45.1 mice, respectively. For serial transplantation, BM cells were collected from all recipient mice at 12–20 weeks after transplantation and pooled together. Then, 5×10^6^ BM cells were transplanted into 8-week-old B6-CD45.1 mice irradiated at a dose of 9.5 Gy without competitor cells. Third and fourth transplantation were similarly performed using 5×10^6^ pooled BM cells. Peripheral blood (PB) cells of the recipient mice were analyzed with a mixture of antibodies that included PE/Cy7-conjugated anti-CD45.1, pacific blue-conjugated anti-CD45.2, PE-conjugated anti-Mac-1 and anti-Gr-1, APC-conjugated anti-B220, and APC/Cy7-conjugated anti-CD4 and anti-CD8α antibodies. Cells were analyzed on a FACS Canto II. Percent donor chimerism was calculated as (% donor cells) ×100/(% donor cells + % recipient cells). To obtain the competitive repopulating units (CRUs), CRU assays were performed with a limiting number of test cells and the data were analyzed using L-Calc software (StemCell Technologies). Peripheral blood cell counts were made using an automated cell counter, Celltec α (Nihon Kohden).

### Apoptosis Analysis

Bulk sorted CD34^-^LSK cells were incubated in SF-O3 supplemented with 50 µM?2-β-mercaptoethanol, 0.2% BSA, 1% GPS, 50 ng/ml SCF, 50 ng/ml TPO for 10 days at 37°C in a 5% CO_2_ atmosphere. At day 10 of culture, the cultured cells were incubated with APC-conjugated anti-Annexin V (BD Pharmingen) and propidium iodide at room temperature for 15 min with protection from light. Analysis was performed on FACS Canto II.

### Immunostaining of γH2A.X

Cells were incubated in a culture medium drop on slide glasses pre-treated with poly-_L_-lysine (Sigma) for 2 hours. After fixation with 2% paraformaldehyde and blocking in 4% sheep serum for 30 min at room temperature, cells were incubated with purified anti-phospho-Histone H2A.X (Ser139) antibody (Cell Signaling Technology) for 12 hours at 4°C. The cells were then washed and incubated with Alexa Flour 555-conjugated anti-rabbit IgG goat polyclonal antibody (Invitrogen) for 60 min at room temperature. DNA was counterstained with 4′,6-diamidino-2-phenylindole (DAPI). Images were taken with a Keyence BZ-9000 fluorescence microscope.

### RT-PCR

Total RNA was isolated using TRIZOL LS solution or TRIZOL solution (Invitrogen) and reverse transcribed by the ThermoScript RT-PCR system (Invitrogen) with an oligo-dT primer. Real-time quantitative polymerase chain reaction (PCR) was performed with an ABI prism 7300 Thermal Cycler (Applied Biosystems) using FastStart Universal Probe Master (Roche). The combination of primer sequences and probe numbers are as follows: for *p16^Ink4a^*, probe #91, 5′-AATCTCCGCGAGGAAAGC-3′, and 5′-GTCTGTCTGCAGCGGACTC-3′; for *p19^Arf^*, probe #106, 5′-GGGTTTTCTTGGTGAAGTTCG-3′, 5′- TTGCCCATCATCATCACCT-3′, and for *Bmi1*, probe #95, 5′-AAACCAGACCACTCCTGAACA-3′ and 5′-TCTTCTTCTCTTCATCTCATTTTTGA-3′.

### Western Blotting

Total cell lysate was resolved by SDS-PAGE and transferred to a PVDF membrane. The blots were probed with a mouse anti-Bmi1 (clone 8A9, kindly provided by Dr. N. Nozaki, MAB Institute, Co. Ltd., Japan), and a horseradish peroxidase-conjugated secondary antibody. The protein bands were detected with an enhanced chemiluminescence reagent (SuperSignal, Pierce Biotechnology).

### Detection of ROS

Cells were stained with an antibody cocktail consisting of biotinylated anti-Gr-1, Mac-1, IL-7Rα, B220, CD4, CD8α, and Ter119 monoclonal antibodies. Cells were further stained with PE-conjugated anti-Sca-1, and PE/Cy7-conjugated anti-c-Kit antibodies. Biotinylated antibodies were detected with APC/Cy7-conjugated streptavidin. After staining with antibodies, cells were incubated with CellROX™ Deep Red Reagent (5 µM, Invitrogen) at 37°C for 30 min with protection from light. Dead cells were eliminated by staining with propidium iodide (1 µg/ml, Sigma). Analysis was performed on a FACS Aria II.

## Supporting Information

Figure S1 **Steady state hematopoiesis in** ***Tie2-Cre;R26StopFLBmi1*** **mice.** (A) Hematopoietic analysis of 10-week-old *Tie2-Cre* and *Tie2-Cre*;*R26Stop^FL^Bmi1* mice. Absolute numbers of CMPs, GMPs, MEPs, and CLPs in bilateral femurs and tibiae (upper panels), total spleen cells and LSK cells in the spleen (middle panel), and total thymic cells, CD4+CD8- cells, CD4-CD8+ cells, and CD4+CD8+ cells in the thymus (lower panels) are shown as the mean ± S.D. (*Tie2-Cre*; n = 8, *Tie2-Cre*;*R26Stop^FL^Bmi1*; n = 7). (B) Cell cycle status of CD34-LSK cells examined by Pyronin Y incorporation. Proportion of CD34-LSK cells in the G0 phase of the cell cycle (Pyronin Y-) was shown as the mean ± S.D. (n = 4) (left panel). Representative flow cytometric profiles are also depicted (right panel).
(EPS)

Figure S2 **Apoptosis and cell cycle status of** ***Tie2-Cre*** **;** ***R26Stop^FL^Bmi1*** **LSK cells in culture.** (A) The proportion of apoptotic cells in the LSK fraction in culture. CD34-LSK cells from *Tie2-Cre* (Control) and *Tie2-Cre*;*R26Stop^FL^Bmi1* (Bmi1) mice were cultured in the SF-O3 serum-free medium supplemented with 50 ng/ml SCF and TPO. At day 10 of culture, apoptotic cells were detected by staining culture cells with anti-Annexin V and propidium iodide (PI). The percentage of Annexin V+PI- apoptotic cells in the LSK fraction is shown as the mean ± S.D. (n = 5). (B) The cell cycle status of LSK cells overexpressing Bmi1. CD34-LSK cells from *Tie2-Cre* (Control) and *Tie2-Cre*;*R26Stop^FL^Bmi1* (Bmi1) mice were cultured in the SF-O3 serum-free medium supplemented with 50 ng/ml SCF and TPO. At day 10 of culture, the cells were incubated with 10 µM BrdU at 37°C for 30 min and then analyzed using a BrdU Flow Kit. Data are shown as the mean ± SD (n = 4).
(EPS)

Figure S3 **Hematopoietic recovery in recipients of** ***Tie2-Cre;R26Stop^FL^Bmi1*** **HSCs after irradiation.** Fresh BM cells from *Tie2-Cre* and *Tie2-Cre;R26Stop^FL^Bmi1* mice (1×107, CD45.2) were transplanted into 8-week-old CD45.1 mice irradiated at a dose of 9.5 Gy without competitor cells. Four months later, the recipient mice were irradiated at a dose of 5 Gy. Changes in the PB cell count were monitored for 4 weeks (A) and the absolute number of BM LSK cells in bilateral femurs and tibiae was examined at 4 weeks post-irradiation (B). Data are shown as the mean ± SD (n = 5).
(EPS)

Figure S4 **ROS levels in** ***Tie2-Cre;R26Stop^FL^Bmi1*** **cells in culture.** Levels of ROS in cells overexpressing Bmi1 in culture. CD34-LSK cells from *Tie2-Cre* (Control) and *Tie2-Cre;R26Stop^FL^Bmi1* (Bmi1) mice were cultured in the SF-O3 serum-free medium supplemented with 50 ng/ml SCF and TPO. Cells from day 11 or 12 of culture were further cultured for 2 days in the presence of 0.2 mM BSO, then levels of ROS in Lin-Sca-1+c-Kit+ cells and Lin-Sca-1low/−c-Kit+ cells were analyzed using CellROXTM Deep Red Reagent. Data are shown as dots and the mean values are indicated by bars (n = 4).
(EPS)
